# The Multimodal MOPr/DOPr Agonist LP2 Reduces Allodynia in Chronic Constriction Injured Rats by Rescue of TGF-β1 Signalling

**DOI:** 10.3389/fphar.2021.749365

**Published:** 2021-10-06

**Authors:** Annamaria Fidilio, Margherita Grasso, Rita Turnaturi, Giuseppe Caruso, Federica Maria Spitale, Nunzio Vicario, Rosalba Parenti, Salvatore Spoto, Nicolò Musso, Agostino Marrazzo, Santina Chiechio, Filippo Caraci, Lorella Pasquinucci, Carmela Parenti

**Affiliations:** ^1^ Department of Biomedical and Biotechnological Sciences, Section of Pharmacology, University of Catania, Catania, Italy; ^2^ Department of Drug and Health Sciences, Section of Pharmacology and Toxicology, University of Catania, Catania, Italy; ^3^ Oasi Research Institute - IRCCS, Troina, Italy; ^4^ Department of Drug and Health Sciences, Section of Medicinal Chemistry, University of Catania, Catania, Italy; ^5^ Department of Biomedical and Biotechnological Sciences, Section of Physiology, University of Catania, Catania, Italy; ^6^ Department of Biomedical and Biotechnological Sciences, Section of Biochemistry, University of Catania, Catania, Italy

**Keywords:** neuropathic pain, transforming growth factor-β1 (TGF-β1), dual target MOPr/DOPr agonist, analgesia, microglia

## Abstract

Neuropathic pain is one of the most disabling forms of chronic pain and it is characterized by hyperalgesia and allodynia linked to an aberrant processing of pain transmission and to neuroinflammation. Transforming growth factor-β1 (TGF-β1) is an anti-inflammatory cytokine, which protects against neuroinflammation. It has been demonstrated that TGF-β1 and opioid receptors signalling crosstalk results in an improvement of endogenous opioid analgesia, but it is not known whether mu opioid peptide receptor (MOPr) or delta opioid peptide receptor (DOPr) agonists can positively modulate TGF-β1 pathway. In the present study, we examined the correlation between anti-allodynic effect of LP2, a dual-target MOPr/DOPr agonist, and TGF-β1 signalling in the chronic constriction injury (CCI) model. We detected a significant decrease of active TGF-β1 and of its type II receptor TGFβ-R2 levels in the spinal cord from CCI rats and a selective deficit of TGF-β1 in microglia cells both at days 11 and 21 post-ligature, as assessed by immunofluorescence analysis. LP2, when administered from the 11 days post-ligature to 21 days, was able to reduce CCI-induced mechanical allodynia by rescue of TGF-β1 and TGFβ-R2 levels. Our data suggest that the rescue of TGF-β1 signalling by dual-target MOPr/DOPr agonist LP2 could be mediated by DOPr activation in spinal microglia, thus the dual-target approach could represent a novel pharmacological approach to increase the analgesic efficacy of MOPr agonists.

## Introduction

Neuropathic pain, one of the most complex and disabling forms of chronic pain, is currently defined as “pain arising as a direct consequence of a lesion or disease affecting the somatosensory system” (Treed et al., 2008). It is often associated with different pathological conditions such as diabetes mellitus, cancer, vascular and infectious diseases. It is characterized by typical symptoms such as an exaggerated pain perception of noxious stimuli (more commonly known as hyperalgesia) as well as non-noxious stimuli (allodynia), linked to an aberrant processing of pain transmission (Costigan, et al., 2009). The underlying mechanisms include complex interactions between neurons, glia, and cells of the immune system through neurotransmitters, cytokines, and inflammatory mediators (Mika et al., 2013), so that neuropathic pain may now be considered as a “neuroimmune disorder” (Malcangio, 2019). Glia cells, which represent about 70% of the cells in the central nervous system (CNS), and microglia activation, with a shift in the balance between pro-inflammatory and anti-inflammatory cytokines, play a key role in sensitization processes (Gwak et al., 2017; Caraci et al., 2019). Pro-inflammatory cytokines such as Interleukin (IL)-1, IL-6, IL-15, IL-17, IL-18, tumor necrosis factor-alfa (TNF-α), and interferon-gamma (IFN-γ) play a key role in the processes of central sensitization, while the induction of anti-inflammatory cytokines [IL-10, IL-4, or transforming growth factor-β1 (TGF-β1)] can exert a protective role against neuroinflammatory events underlying neuropathic pain (Uçeyler et al., 2007; Chen et al., 2013; Caraci et al., 2019). Thus, anti-nociceptive strategies include inhibition of pro-inflammatory cytokines and use of anti-inflammatory cytokines (Shubayev et al., 2010). It has been hypothesized that a deficit of TGF-β1 signalling might contribute to the pathophysiology of chronic pain (Lantero et al., 2012). TGF-β1 can counteract the development of chronic neuropathic pain (Lantero et al., 2014) and exert anti-allodynic and analgesic effects in animal models of neuropathic pain (Echeverry et al., 2009; Lantero et al., 2014). Moreover, TGF-β1 is involved in the pathogenesis of depressive disorders, which often occur in comorbidity with chronic pain (Caraci et al., 2018). Echeverry et al. also observed that recombinant TGF-β1, delivered into the spinal cord of rats, was effective not only in preventing, but also in reversing the hypersensitivity evoked by damage to peripheral nerve through different mechanisms, i.e. blocking microglial cells proliferation, inhibiting spinal microglial activation, and reducing the expression of pro-inflammatory cytokines (Echeverry et al., 2009). Furthermore, Lantero et al. demonstrated that pre- and post-synaptic modulation of the endogenous opioid system by TGF-β1 signalling can prevent the development of allodynia and improve the analgesic efficacy of both endogenous and exogenous opioid agonists both in inflammatory and neuropathic pain conditions (Lantero et al., 2014).

Available therapies with mu opioid peptide receptor (MOPr) agonists often provide incomplete pain relief and treatment-related side effects are common such as respiratory depression, constipation, and tolerance (Chou et al., 2015). A novel drug discovery strategy in chronic pain is the development of MOPr/delta opioid peptide receptor (DOPr) dual-target compounds able to activate MOPr as well as DOPr (Martínez-Navarro et al., 2019). The MOPr/DOPr simultaneous targeting is supported by the co-expression of both receptors in areas involved in pain modulation and by the crucial role of MOPr activation in the regulation of DOPr trafficking (Scherrer et al., 2009). Moreover, activation of DOPr leads to fewer typical opioid side effects.

We previously reported that the dual-target benzomorphan-based compound LP2 was able to simultaneously bind and activate MOPr (K_i_ = 1.08 nM, IC_50_ = 21.5 nM) and DOPr (K_i_ = 6.6 nM, IC_50_ = 4.4 nM) (Pasquinucci et al., 2017). In tail-flick and formalin test LP2 was found to produce significant anti-nociceptive (ED_50_ = 0.9 mg/kg i.p.) and anti-inflammatory (ED_50_ = 0.88 and 0.79 mg/kg i.p., phases I and II of formalin test) effects (Pasquinucci et al., 2019). In rats subjected to unilateral sciatic nerve chronic constriction injury (CCI), we already showed that LP2 significantly inhibits the development of mechanical allodynia and prevents CCI-induced Cx43 alterations and pro-apoptotic signalling in the CNS (Vicario et al., 2019a). These findings prompted us to further examine the molecular mechanisms underlying the analgesic effects of the MOPr/DOPr agonist LP2 in the CCI model. Presently, it is not known whether MOPr and/or DOPr agonists can positively modulate TGF-β1 pathway and whether the rescue of TGF-β1 signalling can contribute to increase the analgesic effects of MOPr/DOPr compounds. Thus, in the present study we examined the hypothesis that CCI can induced an impairment of the TGF-β1 pathway and that the anti-allodynic effects of LP2 in the CCI rat model can be mediated by an increased expression of TGF-β1 and its type 2 receptor (TGFβ-R2) at spinal cord level along the time course of neuropathic pain.

## Materials and Methods

### Animal Model of Neuropathic Pain

Experiments were carried out on male Sprague-Dawley rats (Envigo Laboratories), weighing 180–200 gr. Animals were set at a constant temperature (23–25°C) between 9:00 am and 15:00 pm. This study was performed according to the European Communities Council directive and Italian regulations (EEC Council 2010/63/EU and Italian D. Lgs. no. 26/2014) and approved by Italian Ministry of Health (OPBA Project 946/2018-PR) in order to replace, reduce, and refine the use of laboratory animals. The model used to induce neuropathic pain was the CCI model, according to Bennett and Xie. (1988), with secondary (minor) modifications (Parenti, et al., 2013). Animals were put in a chamber, anesthetized with isoflurane inhalation (4% induction, 2% for maintenance), and an incision was made underneath the hipbone, parallel to the common sciatic nerve, which was exposed. Later, four ligatures (4/0 chromic silk, Ethicon) were tied firmly around the nerve, proximal to the trifurcation of the nerve at about 1 mm spacing, observing a twitch in the respective hind limb. In the SHAM rats, the sciatic nerve was exposed, but there were not made any ligatures. Then, rats were randomly assigned to three different groups: SHAM-vehicle, CCI-vehicle, and CCI + LP2. Starting from 11 days post-ligatures (dpl), after the measurements of allodynic thresholds, CCI groups received a daily intraperitoneal (i.p.) injection of either vehicle or LP2 (0.9 mg/kg) up to 21 dpl.

### Evaluation of Mechanical Allodynia

Allodynia, already measured in the present CCI model in Vicario et al. (2019a), has been reproduced in a new cohort of rats. Rats were allocated and allowed to acclimate for 20 min in a wire mesh bottom test chamber. The ventral surface of the hind paw was mechanically stimulated from below with an ascending series of calibrated Von Frey’s filaments, which bending forces ranging from 0.02 to 30 g. The “up-down” method was used to evaluate the withdrawal threshold, increasing and decreasing sequentially the stimulus strength (Dixon, 1980). Behavioural assessment of mechanical allodynia has been performed at 0 (before surgery), 11, 16 and 21 dpl.

### *Ex vivo* Tissue Processing

At 11, 16, and 21 dpl, rats were anesthetized with an i.p. injection of ketamine (10 mg/ml) and xylazine (1.17 mg/ml), and transcardially perfused with 0.5 M ethylenediaminetetraacetic acid (EDTA) in normal saline, followed by ice cold 4% paraformaldehyde (PFA) in phosphate-buffered saline (PBS) (pH = 7.4) (Sigma-Aldrich, St Louis, MO).

Spinal cords were isolated and post-fixed in 4% PFA in PBS at 4°C overnight. Tissue samples were then washed in PBS and cryo-preserved in 30% sucrose in PBS at 4°C for 3 days. Samples were embedded in optimum cutting temperature (OCT) medium and snap frozen in liquid nitrogen for cryosectioning using a cryostat (Reichert-Jung 2800). Twenty μm-thick axial section of spinal cords from the lumbar enlargement (L4-L5) were collected and stored at −80°C until use.

### RNA Isolation and Quantification

Total RNA was extracted from spinal cord slices (thickness of 20 µm) using the commercial RNeasy FFPE kit (Qiagen, Hilden, Germany) according to the manufacturer’s recommendations, with different modifications carried out in order to improve the yield and purity of the extracted RNA. Briefly, the slides containing spinal cord slices were taken out from the −80°C and left to dry at room temperature (RT). Next, each slide was washed at least three times by using DEPC water and the spinal cord slides were harvested by employing a sterile scalpel and moved to microcentrifuge tubes. After the addition of Buffer PKD, each sample was vortexed, centrifuged, and mixed with proteinase K. Following three different incubation steps (56, 80, and 4°C) and one centrifugation, the supernatant of each sample was transferred to a new microcentrifuge tube. Each tube was therefore added with a mixture of DNase Booster Buffer and DNase I stock and incubated at RT. At the end of the incubation, Buffer RBC and ethanol (100%) were added and the total solution content was moved to the RNeasy MinElute spin column. After two washing steps (Buffer RPE), the RNA was eluted by the addition to the columns of RNase-free water. The concentration of total RNA recovered from spinal cord slices was determined by measuring the fluorescence with Qubit fluorometer (Thermo Fisher Scientific, Waltham, MA, United States) and the absorbance at 260 nm with NanoDrop® ND-1000 (Thermo Fisher Scientific). The first allows more accurate measurements in terms of quantity compared with commonly used methods absorbance-based because the dyes part of the Qubit kit fluoresce only when bound to RNA. The additional measurement with NanoDrop® ND-1000 was carried out to verify the purity of the samples by analyzing the absorbance curves.

### Gene Expression Analysis by Quantitative Real-Time PCR (qRT-PCR)

Gene expression analysis by qRT-PCR was performed as previously described (Caruso et al., 2019) with slight modifications. The reverse transcription of 20 ng of total RNA (for each sample) was accomplished by using the SuperScript III First-Strand Synthesis SuperMix kit (Thermo Fisher Scientific), while the quantification of each cDNA sample loaded in a 384-well plate was achieved by using a LightCycler® 480 System (Roche Molecular Systems, Inc., Pleasanton, CA, United States). The list containing the information of each primer used for this study is reported in Table 1. The protocol employed for sample amplification, fluorescence data collection, and sample quantification is the same previously described elsewhere (Caruso et al., 2019).

**TABLE 1:** The list of primers used for quantitative real-time PCR (qRT-PCR).

Official name^a^	Official symbol	Alternative titles/symbols	Detected transcript	Amplicon length	Cat. No^b^
interleukin 1 beta	Il1b	Il-1b; IL-1beta; IL-1β	NM_008361 XM_006498795	150 bp	QT01048355
				682 bp	
interleukin 6	Il6	Il-6	NM_031168	128 bp	QT00098875
transforming growth factor, beta 1	Tgfb1	Tgfb; Tgfb-1; TGFbeta1; TGF-beta1	NM_011577	145 bp	QT00145250
transforming growth factor, beta receptor 2	Tgfbr2	Tgfbr2T; TGF-beta 2	NM_031132	99 bp	QT00182315
			XM_008766690		

a https://www.ncbi.nlm.nih.gov/gene/

b https://www.qiagen.com/it/shop/pcr/real-time-pcr-enzymes-and-kits/two-step-qrt-pcr/quantitect-primer-assays/

### Western Blot Analysis

To evaluate TGF-β1 and TGFβ-R2 expression levels, the slides containing eight spinal cord slices for each rat, obtained by the procedure described in the *Ex vivo Tissue Processing* paragraph, were washed three times by using deionized water. Next the samples were pooled by employing a sterile scalpel and harvested into microcentrifuge tubes. The samples were resuspended in 3% sodium dodecyl sulfate (SDS) RIPA buffer supplemented by phosphatases and proteases inhibitors (1:100 dilution), incubated for 20 min at 100°C, and for 20 min at 4°C, then were sonicated and subsequently centrifuged for 15 min at 15.000× g in refrigerate centrifuge to remove cellular debris.

Protein quantification was performed using a Pierce™ BCA protein assay kit (Thermo Fisher Scientific), according to the manufacturer’s specifications; subsequently, 30 µg of total proteins were denatured at 95°C for 10 min, subjected to NuPage™ 10% bis–tris gel electrophoresis (Thermo Fisher Scientific) and transferred to nitrocellulose membranes.

The membranes were blotted with anti-TGF-β1 (1:500, Abcam ab92486, Cambridge, United Kingdom), anti-TGFβ-R2 (1:500, Cell signaling Technology Inc., Danvers, MA, United States; 79424), anti-GAPDH (1:2000, Millipore MAB 374, Burlington, MA, United States), and anti-β-actin (1:1000, Sigma Aldrich, St Louis, MO; A4700) primary antibodies in blocking buffer at 4°C overnight. After washing in tris-buffered saline (TBS)/Tween 20 × 0.1%, the membranes were incubated for 1 h with IRDye^®^ 800CW or 680LT secondary antibodies (1:15000) at RT in the dark. Bands were visualized using an Odyssey^®^ infrared imaging system (LI-COR Biosciences, Lincoln, NE, United States), while the densitometric analysis was carried out by using ImageJ software.

### Immunofluorescence

Immunofluorescence was performed as previously described (Gulino et al., 2019; Vicario et al., 2021). Briefly, sections were washed in PBS and incubated with blocking buffer (10% normal goat serum (NGS) and 0.1% Triton X-100 in PBS) for 1 h at RT. Sections were then incubated overnight at 4°C with the following primary antibodies: mouse monoclonal (A60) anti-NeuN (Millipore Cat. No. MAB377, RRID: AB_2298772, 1:100), mouse monoclonal anti-GFAP (BD Biosciences Cat. No. 610566, RRID: AB_397916, 1:100), goat polyclonal anti-IBA1 (Novus Biologicals, Cat. No. NB100-1028, RRID: AB_521594, 1:500), and rabbit polyclonal anti-TGF-β1 (Abcam Cat. No. ab92486, RRID: AB_10562492, 1:100). The following day, sections were washed in 0.1% Triton X-100 in PBS 3 times at RT and then incubated 1 h at RT with appropriate combination of secondary antibodies: goat polyclonal anti-mouse (Alexa Fluor 488, Thermo Fisher Scientific, Cat. No. A-11001, RRID: AB_2534069, 1:1.000), goat polyclonal anti-rabbit (Alexa Fluor 564, Molecular Probes, Cat. No. A-11010, RRID: AB_143156, 1:1.000) and donkey anti-goat (Alexa Fluor 647, Thermo Fisher Scientific, Cat. No. A-21447, RRID: AB_2535864, 1:1.000). Nuclei were counterstained with DAPI (1:10.000, Invitrogen, Waltham, MA, United States) for 5 min at RT and then mounted with BrightMount mounting medium (Abcam). Digital images were acquired using a Leica DM IRB fluorescence microscope and with Leica TCS SP8 confocal microscope and profile plots for immunofluorescence images were obtained as previously described (Vicario et al., 2019a; Spitale et al., 2020). Briefly, confocal images of ipsilateral dorsal horns were analyzed using ImageJ software. For each population marker (i.e. NeuN, Gfap, or Iba1) a profile plot was calculated and superimposed to the corresponding TGF-β1 profile plot in order to highlight proximity and/or colocalization.

### Statistical Analysis

Statistical analysis was performed by using GraphPad Prism 9 (GraphPad Software, La Jolla, CA). Two-tailed unpaired Student’s t-test was used for comparison of *n* = 2 groups. Comparisons of *n* > 2 groups were performed using a one-way analysis of variance (ANOVA). Statistical analyses of behavioral assessment of mechanical allodynia were performed using a two-way ANOVA repeated measure. One-way ANOVA, followed by Bonferroni’s post hoc test, was used for multiple comparisons on molecular markers. Only two-tailed *p*-values of less than 0.05 were considered statistically significant. All data are represented as means ± SEM.

## Results

### The MOPr/DOPr Agonist LP2 Reduces the Mechanical Allodynia in CCI Rats

**FIGURE 1:**
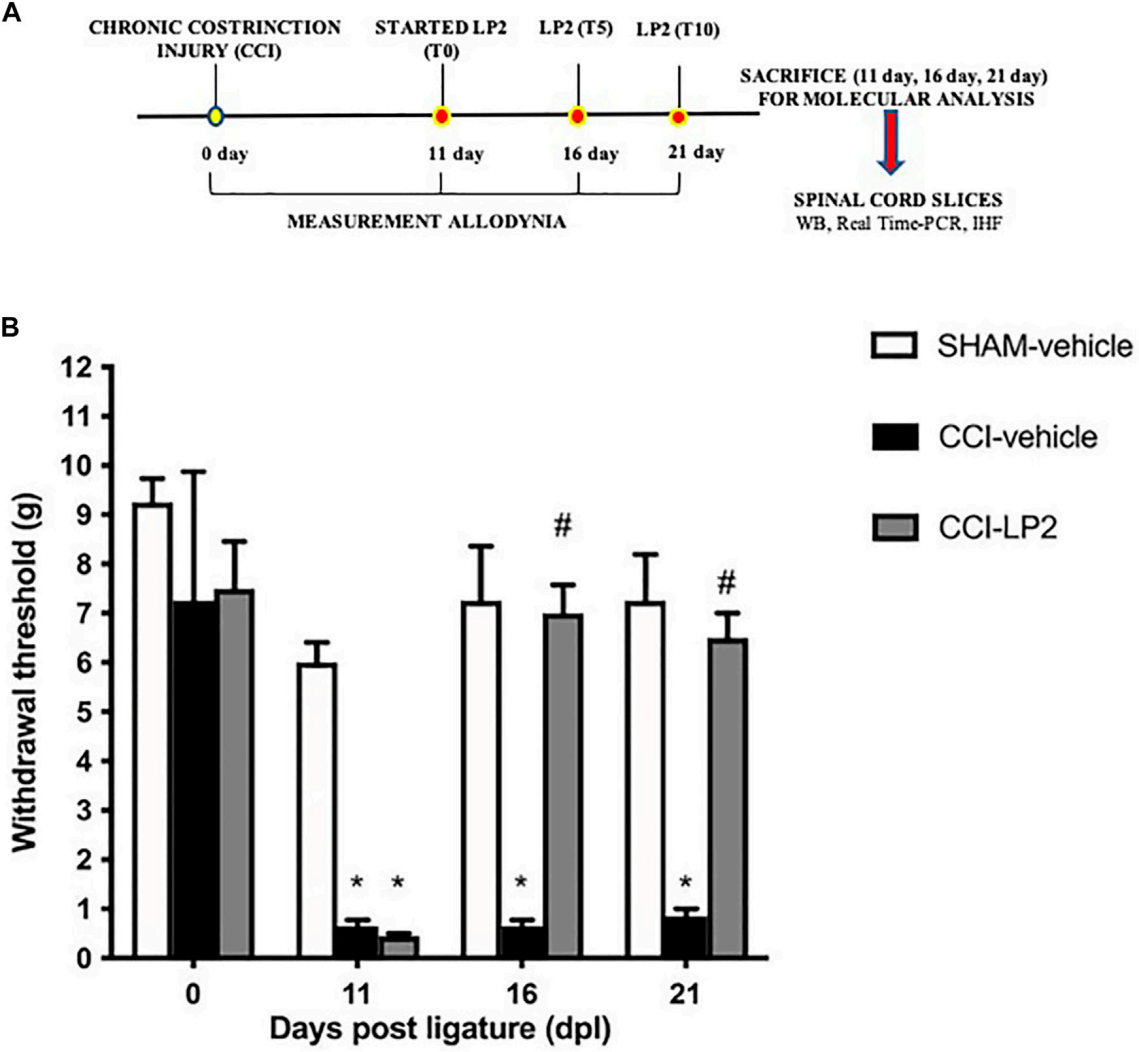
The MOPr/DOPr agonist LP2 shows an anti-allodynic effect in CCI animal model. **(A)** Schematic representation of the experimental design. WB = Western Blot; Real Time-PCR = Real Time Polymerase Chain Reaction; IHF = Immunohistofluorescence analysis. **(B)** Withdrawal thresholds measured with von Frey’s filaments on SHAM-vehicle, on SHAM-vehicle, CCI-vehicle, and CCI + LP2-treated rats at 0, 11 (before the start of LP2 administration), 16, and 21 dpl. Data are shown as mean ± SEM of *n* = 8 rats per group. *p < 0.001 vs SHAM-vehicle. ^**#**^ *p* < 0.001 vs. CCI-vehicle.

We first evaluated the antinociceptive effect of LP2 through the behavioral assessment of mechanical allodynia following the experimental paradigm shown in Figure 1A. Neuropathic pain condition, produced by CCI model, decreased the withdrawal threshold, with a significant reduction at 11 dpl in CCI animals as compared to SHAM rats (Figure 1B). LP2 administration, started from 11 dpl (after withdrawal threshold measurements) recovered the withdrawal threshold at 16 and 21 dpl of CCI + LP2 treated rats as compared to CCI-vehicle rats.

### Molecular Mechanisms Underlying the Anti-allodynic Effects of LP2: The Key Role of TGF-β1

**FIGURE 2:**
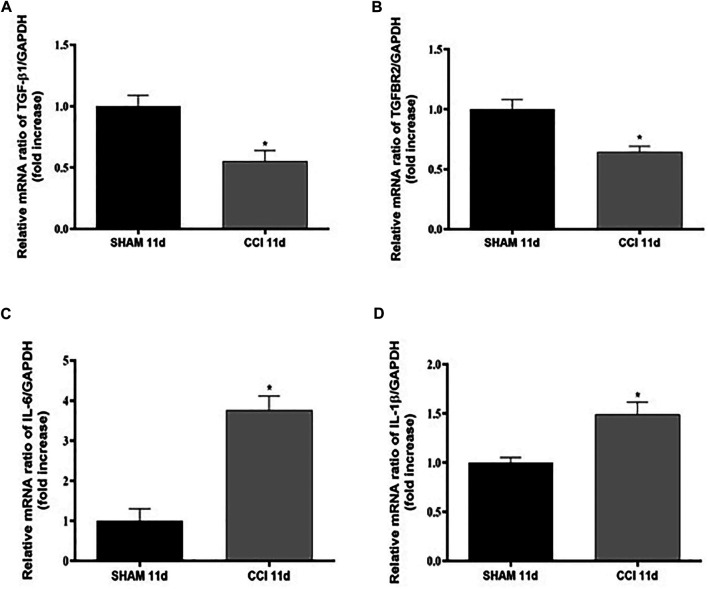
CCI increases pro-inflammatory cytokines levels and reduces TGF-β1 levels 11 dpl. Measurement of **(A)** TGF-β1 (t = 3,593, df = 4; F, DFn, Dfd = 1,031, 2, 2), **(B)** TGFβ-R2 (t = 3,761, df = 4; F, DFn, Dfd = 3,027, 2, 2), **(C)** IL-6 (t = 5,955, df = 6; F, DFn, Dfd = 1,332, 3, 3), and **(D)** IL-1β (t = 3,206, df = 5; F, DFn, Dfd = 6,952, 3, 2) mRNA expression levels (RT-qPCR) in spinal cord of SHAM or CCI rats after 11 days. The abundance of each mRNA of interest was expressed relative to the abundance of GAPDH mRNA, as an internal control. Data are shown as mean ± SEM of *n* = 3–4 rats per group. Statistical analysis was performed using Student’s t-test. *p < 0.05 *vs.* SHAM.

In order to assess the impact of CCI on neuroinflammation in the spinal cord, the gene expression of TGF-β1 and its receptor (TGFβ-R2) along with two well-known pro-inflammatory cytokines, IL-6 and IL-1β, was firstly investigated in the spinal cord at 11 dpl in SHAM and CCI animal groups (Figure 2).

CCI represents a validated animal model of neuropathic pain where neuroinflammation in the spinal cord is known to play a key role in central sensitization (Nong and Lan, 2018). Interestingly we found that CCI significantly decreased the expression levels of TGF-β1 (Figure 2A) and of its receptor TGFβ-R2 (Figure 2B), while raised both IL-6 (Figure 2C) and IL-1β (Figure 2D) mRNA expression levels compared to the rats belonging to the SHAM group (*p* < 0.05 for all cytokines).

**FIGURE 3:**
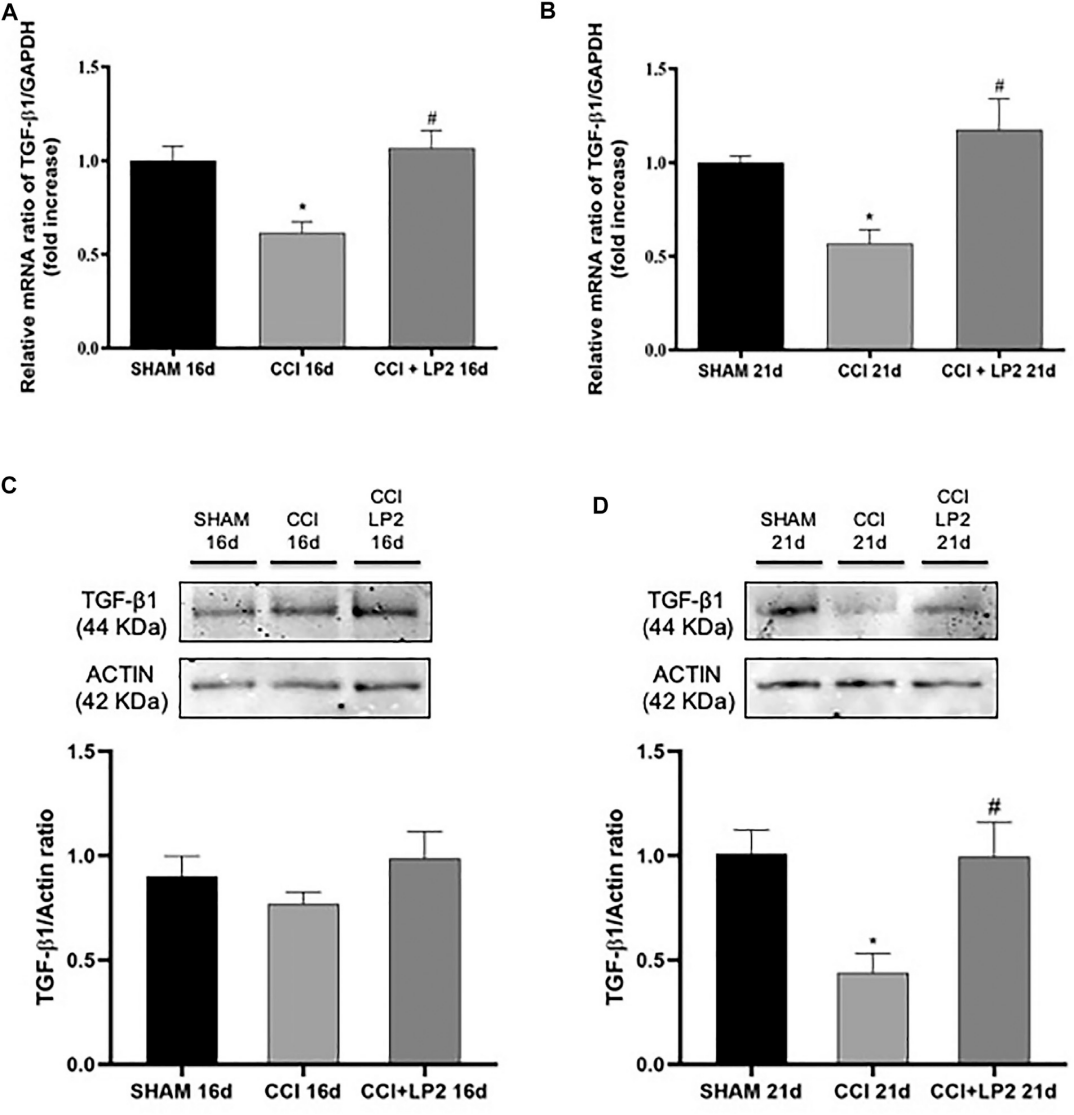
LP2 treatment rescues TGF-β1 levels at 16 and 21 days after chronic constriction injury. Measurement of **(A**–**C)** TGF-β1 at 16 days and **(B**–**D)** TGF-β1 at 21 days mRNA and protein expression levels in spinal cord of SHAM, CCI, and CCI + LP2 rats. **(A**–**B)** The abundance of each mRNA of interest was expressed relative to the abundance of GAPDH mRNA, as an internal control. Data are shown as mean ± SEM of *n* = 3 rats per group. TGF-β1 16 days [F (2, 6) = 10,02]; TGF-β1 21 days [F (2, 7) = 9,961]. **(C**–**D)** Representative immunoblots of TGF-β1 (44 kDa) in total protein extracts from spinal cord tissues at 16 (F (2, 15) = 1,222) and 21 [F (2, 9) = 6,638] dpl. Histograms refer to independent experiments means ± SEM of the TGF-β1 densitometric values normalized against actin used as internal control. Data are shown as mean ± SEM of *n* = 4–6 rats per group. Statistical analysis was performed using one-way ANOVA with Bonferroni’s *post-hoc* analysis. *p < 0.05 *vs.* SHAM; #*p* < 0.05 *vs.* CCI.

**FIGURE 4:**
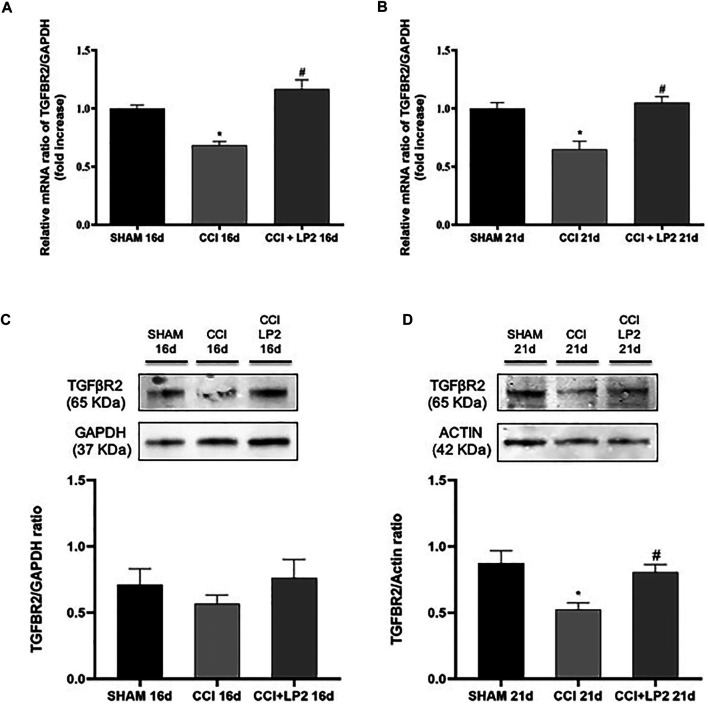
CCI induces a reduction of TGFβ-R2 mRNA and protein expression levels reversed by a chronic treatment with LP2. Effects induced by CCI procedure in the absence or presence of LP2 treatment at 16 and 21 dpl on TGFβ-R2 levels evaluated by RT-qPCR and western blot analysis. **(A**–**B)** The abundance of each mRNA of interest was expressed relative to the abundance of GAPDH mRNA, as an internal control. Data are shown as mean ± SEM of *n* = 3 rats per group. TGFβ-R2 16 days [F (2, 7) = 22,66]; TGFβ-R2 21 days [F (2, 5) = 13,51]. **(C**–**D)** Representative immunoblots of TGFβ-R2 (65 kDa) in total protein extracts from spinal cord tissues at 16 [F (2, 12) = 0,8078] and 21 [F (2, 12) = 7,197] dpl. Histograms refer to independent experiments means ± SEM of the TGFβ-R2 densitometric values normalized against GAPDH and actin used as internal controls by one-way ANOVA with Bonferroni’s *post-hoc* for statistical analysis. Data are shown as mean ± SEM of *n* = 4–6 rats per group. *p < 0.05 *vs.* SHAM, #*p* < 0.05 *vs.* CCI.

To understand the molecular mechanisms underlying the anti-allodynic effects of LP2 in CCI rats, we then assessed whether LP2 exerted its analgesic effects in our animal model of neuropathic pain by counteracting neuroinflammatory phenomena induced by CCI (SHAM *vs.* CCI *vs.* CCI + LP2) after 5 and 10 days of LP2 treatment, respectively (i.e. at 16 and 21 dpl) (Figures 3, 4). Figures 3A,B clearly shows that the significant decrease in mRNA expression levels of TGF-β1 induced by CCI, still persisting after 16 and 21 days (*p* < 0.05 *vs* SHAM). The same effect was observed for the TGFβ-R2 gene expression at 16 and 21 dpl (Figures 4A,B) (*p* < 0.05 *vs*. SHAM).

Interestingly we found that the treatment with LP2 completely rescued both TGF-β1 (Figures 3A,B) and TGFβ-R2 (Figures 4A,B) mRNA levels in CCI rats (*p* < 0.05 *vs.* CCI) after 5 and 10 days of treatment. Unlike TGF-β1 and TGFβ-R2, the treatment with LP2 was not able to counteract the increased production of IL-6 and IL-1β CCI-induced (data not shown).

TGF-β1 activity in the CNS is regulated not only at a transcriptional level, but it shows a complex post-translational regulation through the conversion of latent TGF-β1 to active TGF-β1 by a variety of proteases, among which matrix metalloproteinase 2 (MMP-2) and matrix metalloproteinase 9 (MMP-9) play a central role in this conversion (Caraci et al., 2018).

To validate the role of TGF-β1 as a new pharmacological target in neuropathic pain, we therefore quantified the protein levels of active TGF-β1 and its receptor (TGFβ-R2) in the spinal cord of CCI rats both in the absence and in the presence of LP2 treatment. Interestingly, we found that CCI procedure was able to induce a significant decrease of active TGF-β1 (Figures 3C,D) and TGFβ-R2 levels (Figures 4C,D) only 21 dpl (*p* < 0.05 *vs.* SHAM), but not at 16 dpl. Most importantly, LP2 treatment was able to rescue both TGF-β1 and TGFβ-R2 levels when compared with those in CCI rats (*p* < 0.05 *vs.* CCI; Figures 3C,D; Figures 4C,D), suggesting that only a long treatment with LP2 is able to rescue the TGF-β1 pathway in CCI rats.

### The Multimodal MOPr/DOPr Agonist LP2 Induces an Increase of TGF-β1 Which Colocalizes With Iba1 in CCI Rats

**FIGURE 5:**
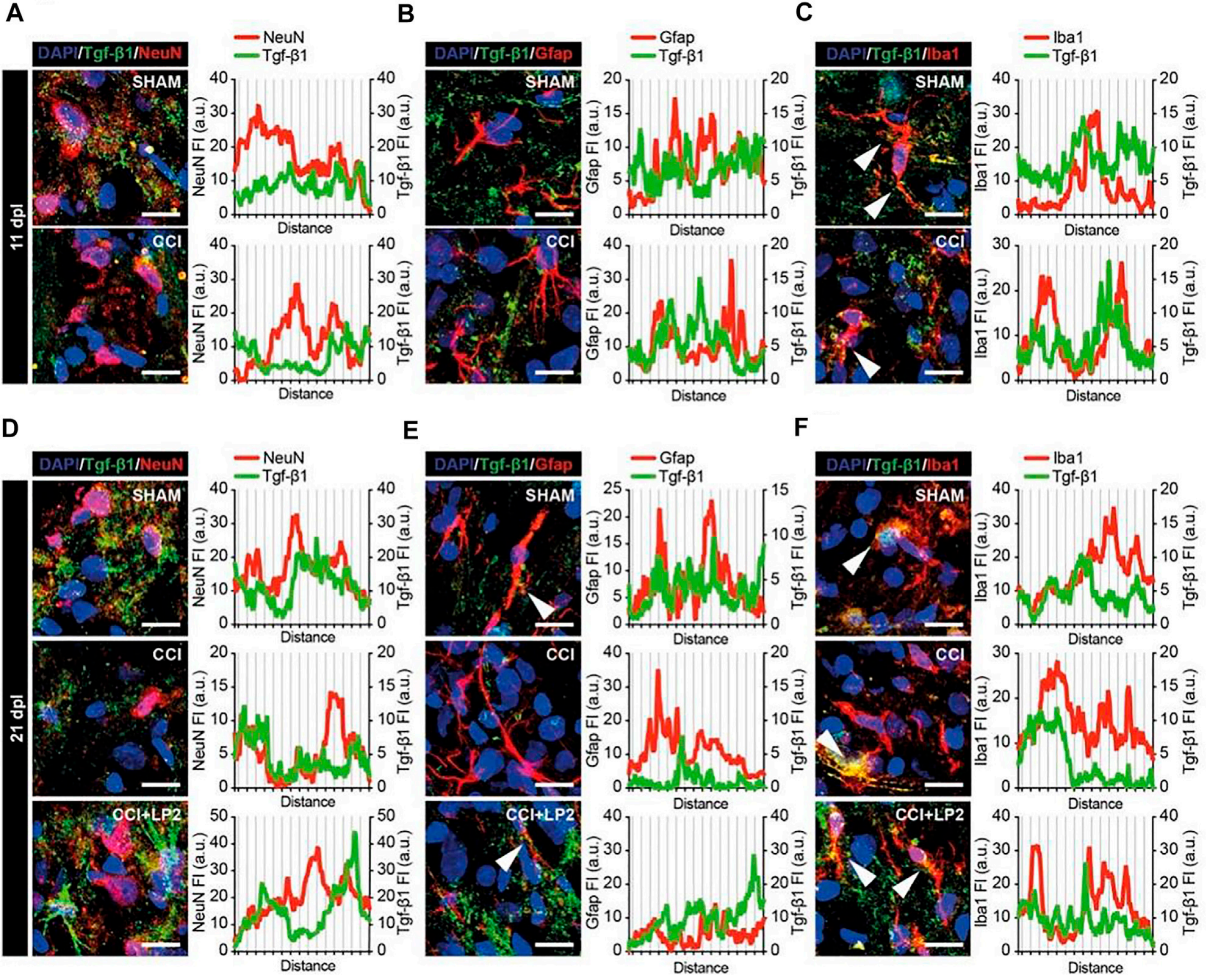
TGF-β1 profile in neurons, astrocytes and microglial cells in ipsilateral dorsal horns of CCI spinal cord. Representative confocal images of TGF-β1 (green) and NeuN **(**red in **A**–**D)**, GFAP **(**red in **B**–**E**) and Iba1 **(**red in **C**–**F)** immunohistofluorescence analysis in ipsilateral dorsal horns of SHAM and CCI rats at 11 dpl **(A**–**C)** and of SHAM, CCI and CCI + LP2 treated rats at 21 dpl **(D**–**F).** Scale bars 10 μm. Profile plots of mean fluorescence intensity, expressed as arbitrary units (a.u.), are also shown.

To evaluate the expression and localization of TGF-β1 in CCI model, we analyzed ipsilateral dorsal horns cell population (i.e., NeuN positive neurons, GFAP positive astrocytes, and Iba1 positive microglial cells) and their potential colocalization or proximity to TGF-β1 in the early phase of the neuropathy (Figures 5A–C). We observed that CCI induced an overall reduction of TGF-β1, which was found to be in close proximity to NeuN positive cells (Figure 5A) and to colocalize with Iba1 positive cells (Figure 5C). These data demonstrate a selective deficit of TGF-β1 in the spinal microglia in the CCI model. Such a deficit was also observed in the chronic stage of CCI (i.e., 21 dpl, Figures 5D–F). Indeed, profile plot analysis revealed that LP2 treatment was able to increase peri-neuronal TGF-β1 signals (Figure 5D), which weakly colocalize with NeuN (Figure 5D) and GFAP (Figure 5E) positive cells. Interestingly we found a strong colocalization between TGF-β1 and Iba1 positive cells (Figure 5F), thus indicating a critical role of microglial cells in the rescue of TGF-β1 mediated by LP2.

## Discussion

Neuropathic pain represents a chronic pathological condition with a significant negative impact on a patient’s quality of life with a prevalence among the general population ranging from 3 to 17% (Cavalli et al., 2019). Since the typical symptoms of this disease are often unresponsive to conventional therapy, new pharmacological targets have to be identified to improve current therapeutic approaches. In this scenario, the CCI model represents a validated animal model of neuropathic pain useful to identify the molecular mechanisms underlying chronic pain development as well as novel pharmacological targets for its management (Boccella et al., 2018; Coraggio et al., 2018; Caraci et al., 2019).

DOR represents a novel pharmacological target in the treatment of chronic neuropathic pain (Kabli and Cahill, 2007; Cahill et al., 2020). Recent studies have demonstrated that DOPr agonists counteract and prevent nociceptive behaviors in various chronic pain models, including neuropathic pain, while having minimal effect on sensory thresholds in the absence of injury (Turnaturi et al., 2019). We have previously demonstrated that the multimodal MOPr/DOPr agonist LP2 produced a significant anti-nociceptive and anti-inflammatory effect in tail-flick and formalin test, respectively (Pasquinucci et al., 2017; Pasquinucci et al., 2019). Moreover, the repeated administration of LP2 significantly inhibited the development of mechanical allodynia in neuropathic rats subjected to CCI and prevented CCI-induced Cx43 alterations and pro-apoptotic signalling in the CNS (Vicario, et al., 2019a; Vicario, et al., 2019b). Despite selective MOPr agonists are the cornerstones of moderate-to-severe acute pain treatment, their effectiveness and safety in chronic pain conditions is controversial. In contrast to MOPr, DOPr density and activity are up-regulated in chronic pain models and analgesic effects of DOPr selective agonists are enhanced during persistent inflammation (Turnaturi et al., 2019). Moreover, the activation of MOPr plays a crucial role in the regulation of the trafficking and membrane targeting of DOPr in conditions of persistent inflammation (Gendron et al., 2007). Direct coupling of MOPr-DOPrs in the form of hetero-oligomers has been demonstrated in the spinal cord tissue and it was proposed to underlie the anti-nociceptive synergy between MOPr and DOPr agonists (Gomes et al., 2004). Thus, simultaneous activation of MOPr and DOPr with a dual target compound delivers potent analgesia in experimental models of chronic pain with strongly reduced dependence and tolerance development on the course of repeated administration (Starnowska-Sokół and Przewłocka, 2020). Nevertheless, the molecular mechanisms underlying the analgesic effects of DOPr agonists are still unclear as well as the different contribute of glial cells and neurons in the allodynic effects of these compounds. It is known that prolonged morphine treatment *in vivo* induces the translocation of DOPrs from intracellular compartments to neuronal plasma membranes (Hack et al., 2005). DORs are also strongly expressed both in astrocytes and microglial cells (Turchan-Cholewo et al., 2008), but it is presently unknown the contribute of glial DORs in the analgesic effects of DOPr agonists.

Recent studies have demonstrated that the activation of glial cells, in particular microglia, toward a pro-inflammatory state plays a central role in the transition from acute to chronic pain (Coraggio et al., 2018; Caraci et al., 2019; Myers et al., 2006), also contributing to a state of opioid analgesic tolerance (Holdridge et al., 2007). In particular, peripheral damage and hyperactivity of primary sensory neurons promote neuroinflammation, in the spinal cord, through the release of several pro-inflammatory cytokines (e.g., TNF-α and IL-1β) by both activated astrocytes and microglial cells (Ji et al., 2014; Lees et al., 2015), finally leading to central sensitization and pain chronicization (Ji et al., 2014). Interestingly, the repeated activation of DOPr reduces the release of pro-inflammatory cytokines, such as TNF-α (Vicario et al., 2016), suggesting an impact of DOPr agonists on neuroinflammation in animal models of neuropathic pain. Along this line, an alternative approach that has been recently adopted in drug discovery in neuropathic pain is to develop new compounds able to promote the release of anti-inflammatory cytokines endowed with a neuroprotective and analgesic efficacy (Guo et al., 2021; Piotrowska, et al., 2016). Among microglial anti-inflammatory cytokines, TGF-β1 has a protective role against the development of chronic neuropathic pain by inhibiting neuroinflammation and promoting the expression of endogenous opioids within the spinal cord (Lantero et al., 2012). TGF-β1 pathway has been proposed as a novel pharmacological target to increase the analgesic effects of opioids (Onichtchouk et al., 1999; Lantero et al., 2014; de la Puerta et al., 2019). In addition, a TGF-β1-opioid receptor signalling crosstalk results in improvement of endogenous and exogenous opioid analgesia in experimental models of neuropathic pain (Lantero et al., 2012; Lantero et al., 2014).

Starting from this evidence, we therefore examined the impact of CCI on mRNA levels of pro-inflammatory (IL-1β and IL-6) and anti-inflammatory (TGF-β1) cytokines in the spinal cord observing that CCI procedure increased IL-1β and IL-6 levels at 11 days. These results are in accordance with previous findings showing a TNF-α induction in the same model in the sciatic nerve (Vicario et al., 2016). Previous works have also demonstrated an induction of IL-1β and IL-6 in affected nerves from CCI models (Khan et al., 2018; Liu et al., 2020). To the best of our knowledge our data demonstrate for the first time an induction of these cytokines in the spinal cord of CCI rats which co-occurs with a reduction of the expression levels of TGF-β1 and TGFβ-R2 mRNAs 11 dpl compared with SHAM control animals. Interestingly, in our model a significant decrease of both active TGF-β1 levels and TGFβ-R2 levels was detected only 21 dpl, suggesting a long-term and post-transcriptional regulation of TGF-β1 and its receptor in the CCI model or alternatively a selective deficit of them in sub-population of cells from spinal cord (microglial cells vs astrocytes/neurons; see below). TGF-β1 plays a protective role against the development of chronic neuropathic pain and the first demonstration of the anti-nociceptive effects of TGF-β1 has been obtained by Chen et al. (2013), with intrathecal administration of this peptide able to reduce both spinal neuroinflammation and excitoxicity (Chen et al., 2013). In the same animal model, it has been demonstrated that the down-regulation of p38 and extracellular signal-regulated kinase (ERK) activity influences TGF-β1-induced analgesia during neuropathy (Chen et al., 2016). Different groups have analyzed the neurobiological links between TGF-β1 and opioid system in animal models of neuropathic pain (Tramullas et al., 2010; Lantero et al., 2014). Increased levels of a transmembrane pseudo-receptor structurally similar to TGF-β type I receptors, BAMBI (bone morphogenetic protein and activin membrane-bound inhibitor), and TGF-β receptors have been detected in areas with a high density of opioid receptors such as the cingulate cortex, periaqueductal grey matter, and the dorsal horns of the spinal cord, key areas of the inhibitory modulation of pain transmission (Tramullas et al., 2010). An enhancement of TGF-β1 signalling has been observed in mice lacking the TGF-β pseudo-receptor BAMBI, which leads to an increased synaptic release of opioid peptides and to a naloxone-reversible hypoalgesic/antiallodynic phenotype. In in vivo neuropathic pain models, the pleiotropic protective effects of TGF-β1 are achieved by the inhibition of neuroinflammatory phenomena (Echeverry et al., 2009; Chen et al., 2015), suppression of the neuronal hyperexcitability (Chen et al., 2013) and endogenous opioid system activation (Onichtchouk et al., 1999; Lantero et al., 2014; de la Puerta et al., 2019). Nevertheless, all these studied have not deeply investigated the possible presence of a TGF-β1 signalling deficit in the CCI model.

In the present work we demonstrated a selective deficit of TGF-β1 and its receptor in microglia cells from spinal cord of CCI rats detectable both at days 11 and 21 dpl as assessed by immunofluorescence analysis. Previous studies have found conflicting data on TGF-β1 levels in the CCI model, with reduced expression of this factor in dorsal root ganglion from CCI mice, followed by an increase at day 10 post-ligature (Xie et al., 2019) or reduced levels of TGF- β1 and its type I receptor in the red nucleus of rats 2 weeks after spared nerve injury (SNI) (Wang et al., 2015). Further studies are needed in the rat model of CCI to understand whether the selective deficit of TGF-β1 and/or its receptor in microglia can be detected in an early phase of pain chronicization.

It is well-known that a 2 weeks subcutaneous infusion with recombinant TGF-β1 showed an anti-allodynic effect when compared with saline in mice subjected to sciatic nerve injury (Lantero et al., 2012), but alternative and more feasible approaches should be developed to rescue TGF-β1 levels in animal models of neuropathic pain. In the present work we demonstrated, for the first time, that a chronic treatment (10 days) with the multimodal MOPr/DOPr agonist LP2 was able to rescue the levels of active TGF-β1 and of TGFβ-R2 in the spinal cord from CCI rats and, most importantly, in microglial cells. Interestingly Reddy et al. found that activation of DOR in transplanted mesenchymal stem cells with the synthetic peptide (D-Ala^2^, D-Leu^5^)-enkephalin (DADLE) promotes the secretion of anti-inflammatory cytokines (IL-10/IL-4/TGF-β) (Reddy and Sen, 2017). Further studies are therefore needed, in primary cultures of microglial cells, to analyze the effects of LP2 as well as other DOPr agonists on TGF-β1 synthesis. A recent study, conducted in a neuropathic pain rat model generated through spinal nerve ligation (SNL), demonstrated that valproate mitigates SNL-induced allodynia by modulating microglial function, inhibiting neuroinflammatory response, finally promoting the expression of anti-inflammatory cytokines (TGF-β, IL-10 and IL-4) (Guo et al., 2021).

Our study, conducted in a validated animal model of neuropathic pain, is the first to demonstrate that the dual target MOPr/DOPr agonist LP2, when administered from the 11 dpl to 21 days, is able to reduce CCI-induced mechanical allodynia by rescue of TGF-β1 and TGFβ-R2 levels. Moreover, our results show the possible role played by microglial cells in modulating the rescue of TGF-β1 levels promoted by LP2 treatment. We believe that the rescue of TGF-β1 signalling by dual-target MOPr/DOPr agonist LP2 could be mediated by DOR activation in spinal microglia and might represent a novel pharmacological approach to increase opioid analgesic efficacy of selective MOPr agonists.

## Data Availability

The raw data supporting the conclusions of this article will be made available by the authors, without undue reservation.
